# Towards generalizable Federated Learning in medical imaging: A real-world case study on mammography data

**DOI:** 10.1016/j.csbj.2025.03.031

**Published:** 2025-03-20

**Authors:** Ioannis N. Tzortzis, Alberto Gutierrez-Torre, Stavros Sykiotis, Ferran Agulló, Nikolaos Bakalos, Anastasios Doulamis, Nikolaos Doulamis, Josep Ll. Berral

**Affiliations:** a School of Rural, Surveying and Geoinformatics Engineering, National Technical University of Athens, Heroon Polytechneiou 9, Athens, 15773, Attica, Greece; b Barcelona Supercomputing Center, Plaça d'Eusebi Güell, 1-3, Les Corts, Barcelona, 08034, Catalunya, Spain; c Universitat Politècnica de Catalunya, Barcelona Tech, Carrer Jordi Girona, 1-3, Les Corts, Barcelona, 08034, Catalunya, Spain

**Keywords:** Federated Learning, Medical imaging, Privacy preservation, Mammography, Deep learning, BIRADS classification

## Abstract

Federated Learning has been rapidly gaining in popularity in medical applications, due to the increased privacy offered, since medical data doesn't need to leave the hospitals' premises for AI model training. However, a direct translation of a classic experiment to a federated one is not always straightforward. In this work, we delve into the intricacies of federated learning for a breast cancer classification tool. We compare classic model training with a federated variant, and highlight the adaptations that need to be taken care of to ensure the equivalence between the two. Specifically, we introduce the Breast Area Detection tool as an essential component of the pre-processing pipeline to enhance the robustness of Federated Learning by offering data harmonization. On top of that, we present an end-to-end Federated Learning framework that is effective for real-world data and scenarios. Among the three real-world hospitals involved in the experimental procedure, the proposed framework significantly improves performance at the first hospital, providing consistent results similar to those achieved in the classic approach. Experimental results demonstrate that the interventions introduced improved model performance by approximately 35%, aligning federated learning and centralized model performance.

## Graphical abstract

**Figure:**
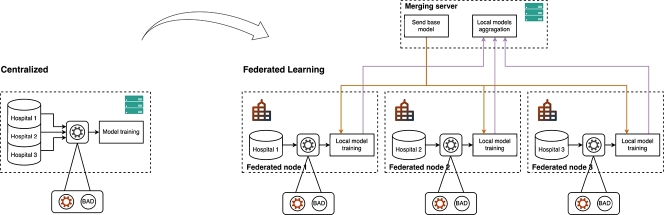

## 1 Introduction

Healthcare is one of the domains that is heavily dominated by human decision making. Although the critical nature of healthcare signifies the need for human expertise, the heavy reliance on human interpretation and assessment creates bottlenecks in the clinical workflow, leading to issues such as delays in the examination process or significant workload on medical professionals (MP) [1]. Therefore, there is an increasing need for automated tools to assist medical professionals in the clinical workflow. The recent rise of Artificial Intelligence (AI) creates unprecedented opportunities for the design and development of such features [2], [3], [4], [5]. It can even be said that AI holds the key to unlocking the untapped potential of software-based medical applications, which would automate certain steps in the clinical workflow or help clinicians by boosting their productivity.

However, the development of AI algorithms requires centralized access to large amounts of data to ensure generalization, which, in the case of medical applications, comes in conflict with privacy concerns, as well as regulations (e.g. GDPR). To that end, researchers have been investigating techniques which can alleviate these privacy issues. One promising technique in the healthcare domain is Federated Learning, which is a machine learning approach where multiple distributed devices collaboratively train a shared model [6], [7]. In a Federated Learning Framework, the data do not need to be gathered in a centralized entity. Instead, in the context of medical applications, sensitive patient data can stay within a hospital's premises. A copy of the model will be transferred to the hospital, trained on the local data, and returned to an external server, which will be responsible for merging the models from different hospitals in an optimal way.

Even though Federated Learning (FL) appears promising on paper, the adaptation of AI workflows to a decentralized architecture is not straightforward. Limited or no access to the training data requires assumptions about their intrinsic properties, statistical distributions, etc. In addition, the training process is hard to debug if the initial results are not as desired. Data-related phenomena, such as data heterogeneity or domain shift, can significantly impact the model training procedure, while being hard to identify. Finally, FL entails increased implementation complexity, as well as model aggregation challenges [8], [9], [10].

This paper highlights the challenges and adaptations required to translate a centralized medical AI model training workflow to a federated one. More specifically, we contend that, by placing emphasis on data preparation and reliable communication mechanisms, even simple aggregation methods can achieve appropriate performance in a FL setting. We utilize the training procedure of a BIRADS classification model on mammography data as a case study to demonstrate the steps that need to be followed to transition from classic training, to a federated variant with similar convergence bounds. Our approach is implemented using three hospitals as federated nodes (two in Greece and one in Serbia) and highlights the lessons learned, as well as suggests mitigation measures for issues encountered. The main outcomes of this work are summarized as follows:

• Pre-processing: the significant variance in the performance of the initial federated learning approach among different data providers, compared to the classic approach, led us to assume that this discrepancy was due to data heterogeneity. To address this, we conceived the idea of building a pipeline specifically targeted at this issue. This pipeline goes beyond traditional methods of medical image processing by introducing the Breast Area Detection tool, a supervised learning-based software designed to remove annotation labels and other artifacts from mammograms.
• Adapting the classic experiment to a federated scheme appeared to be a straightforward solution to critical issues in the medical domain, such as privacy preservation. However, in this work, we demonstrate that the adaptation is not as simple as it seems, particularly when dealing with real-world data and hospitals. Ultimately, we propose an end-to-end framework that performs effectively in this demanding context.
• Lessons learned from the experimentation: A major contribution of this work is the valuable experience gained on this topic, with conclusions that can guide similar efforts. For each observation/lesson, we propose specific mitigation actions and link them to the FUTURE-AI [11] initiative.

### 1.1 Literature review

Artificial Intelligence (AI) has driven significant advancements in healthcare, particularly in medical imaging for cancer diagnosis and prognosis. Among these, deep learning models like Mirai and AsymMirai have demonstrated exceptional capabilities. Mirai, for instance, predicts breast cancer risk up to five years in advance based on mammography data, while AsymMirai emphasizes interpretability by incorporating bilateral asymmetry features, making it more transparent and clinician-friendly [12], [13].

There are several publications related to the classification of BIRADS scores using mammograms to train deep learning models. In [14], the authors introduce the RN-BCNN model, a customized version of the typical ResNet, for classifying mammograms according to the BIRADS scale. A more complex approach is presented in [15], where Transfer Learning principles are adopted to enhance classification results by incorporating state-of-the-art networks such as NASNet Mobile, VGG16, and VGG19. Both studies apply augmentation techniques to expand the limited size of the INbreast dataset. Another noteworthy study, presented in [16], demonstrates the use of a DNN-based model for lesion localization while incorporating the corresponding BIRADS score. Additionally, a two-stage pipeline is introduced in [17], where a YOLOv5-CBAM model detects regions of interest containing lesions, which are then segmented and classified according to the BIRADS score in the second stage.

However, classic centralized AI approaches in healthcare face numerous challenges. Privacy concerns and stringent regulations like HIPAA and GDPR restrict the aggregation of patient data in centralized repositories. Additionally, data heterogeneity—caused by variations in imaging protocols, equipment, and patient demographics—further complicates the development of robust, generalizable models [18], [19]. The inability to effectively harmonize datasets across institutions often leads to biased models that fail to perform well in diverse clinical settings.

Federated Learning (FL) has emerged as a promising solution to these challenges. FL enables collaborative training of AI models while ensuring that sensitive data remains localized. This paradigm has been successfully applied in various medical imaging tasks. For example, Bakas et al. demonstrated the feasibility of FL in brain tumor segmentation, enabling multi-institutional collaboration without compromising patient confidentiality [20], [21], while [22] performed an extensive evaluation in clinical settings, and highlighted the need and ongoing considerations to address security and privacy issues. Similarly, FL has been employed in histopathology image analysis for the classification of tumor-infiltrating lymphocytes, further showcasing its versatility in addressing diverse medical imaging problems [23].

Despite its promise, FL presents its own set of challenges. The non-IID (non-Independent and Identically Distributed) nature of medical imaging data, where datasets from different institutions exhibit significant variability, poses a substantial hurdle to model convergence and generalization. Advanced techniques like FedProx and FedMA have been proposed to address these issues by improving model aggregation and accommodating heterogeneity in training data [24], [25]. Furthermore, FL systems face challenges related to communication overhead and system heterogeneity, where variations in computational resources and infrastructure across participating institutions complicate implementation [19], [26].

A critical area of focus in FL is data harmonization, which seeks to reduce variability across datasets and enhance model generalizability. Studies have highlighted the importance of metadata-driven pre-processing and domain adaptation techniques to align data distributions [18], [27]. The application of Kubernetes-based architectures in FL frameworks has also been notable, enabling scalability and adaptability in real-world implementations [26], [27].

Our work advances the state of the art in Federated Learning (FL) for medical imaging by addressing critical challenges identified in previous studies. Building on the importance of harmonizing heterogeneous datasets as emphasized by Kilim et al. [18] and Zhou et al. [27], we introduce a dynamic harmonization pipeline that adapts pre-processing to variations in imaging protocols and patient demographics. While methods like FedProx [24] and FedMA [25] aim to tackle non-Independent and Identically Distributed (non-IID) data, they have several limitations. Fedprox adds a proximal term to the local training objective of each node, which discourages drastic deviations from the global model. Even though this approach helps in addressing data heterogeneity between nodes, the model may struggle to generalize in the case where not many nodes are participating in model training. This is usually the case in cross-silo scenarios, where the number of participants in FL is limited. FedMA follows a different approach, where layer-wise matching is introduced to align neurons between nodes based on their similarity. This leads to an adaptive model structure that considers data heterogeneity. However, this approach entails significant additional computational/communication complexity in training procedure, as nodes need to implement additional information exchange rounds with the aggregation server. In addition, FedMA only supports model architectures comprising of convolutional and fully connected layers, and layer-wise matching cannot be implemented in building blocks such as Long-Short Term Memory (LSTM) and Transformer layers. These factors restrict its practicality in federated medical settings. In our approach, we argue that more emphasis should be put on data preparation and robust communication mechanisms rather than sophisticated aggregation methods, as some of the cases can be solved by doing so. We use BIRADS classification on mammograms as a case study, and implement a Federated Learning procedure across three hospital institutions from the INCISIVE project (Athens, Novi Sad, Thessaloniki). Our work highlights the steps that need to be followed for the successful implementation of an FL infrastructure, as well as highlights important lessons learned and proposes mitigation measures.

## 2 Context and modeling methodology overview

### 2.1 Dataset and platform details

The work described in this paper has been conducted under the context of the INCISIVE European Project.^2^ This has allowed us to access multiple datasets from different medical institutions, and in particular for this work, mammograms. Data collection was jointly organized with the medical institutions associated to the INCISIVE project. In order to facilitate the data collection and to later use the data, a Joint Controller Data Sharing agreement was put in place. All data were gathered in a common cloud and also stored at the hospital level. An extensive description about the data collection process can be found in the work by Lazic et al. [28], covering both technical and legal aspects of the process. While the FL infrastructure was not ready, the teams developing Artificial Intelligence (AI) models such as the one mentioned in this work, used the Cloud to train their models in the classical setup.

**Fig. 1:**
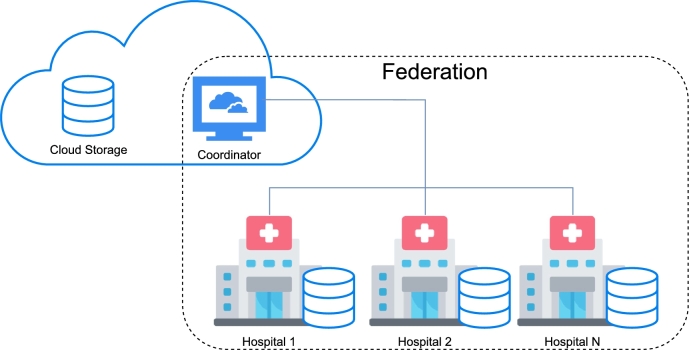
The INCISIVE Project Cloud and Federation infrastructure.

On the other hand, most of the medical institutions had their own on-premise servers where the data was also stored in order to provide an infrastructure for the FL framework. When the medical institution could not set up their own server due to the lack of IT personnel or other issues, a virtual machine was set up in the Cloud for them. In the case of our work, all the institutions had their own on-premise servers. All the machines met the minimum requirements set which were 12th generation Intel i9, AMD Ryzen 5900X or better, 64 GB of RAM and, if possible, a Graphics Processing Unit (GPU) with at least 10 GB of RAM. A detailed description of the implemented FL framework is presented in Section 3.1. The INCISIVE FL framework will serve as the backbone for the EUCAIM 3 project, which aims to serve as the main platform for european federated research in healthcare. The final infrastructure is depicted in Fig. 1, where there are two main parts, the cloud which contained the copy of all data, and the federation which contained the nodes from each hospital as mentioned before. Whenever we make reference to FL, we refer to use the federation in an FL setting, not a simulation in the cloud.

### 2.2 Datasets description

In order to train the BIRADS classification model, we utilized data from three different hospital institutions; Hellenic Cancer Society, University of Novi Sad and Aristotle University of Thessaloniki. It should be noted that the former provided data from multiple hospital institutions, whereas the latter two had direct access to data from the hospital's university clinic. Throughout this work, these entities will be called Hospital 1, Hospital 2 and Hospital 3 respectively.

**Fig. 2:**
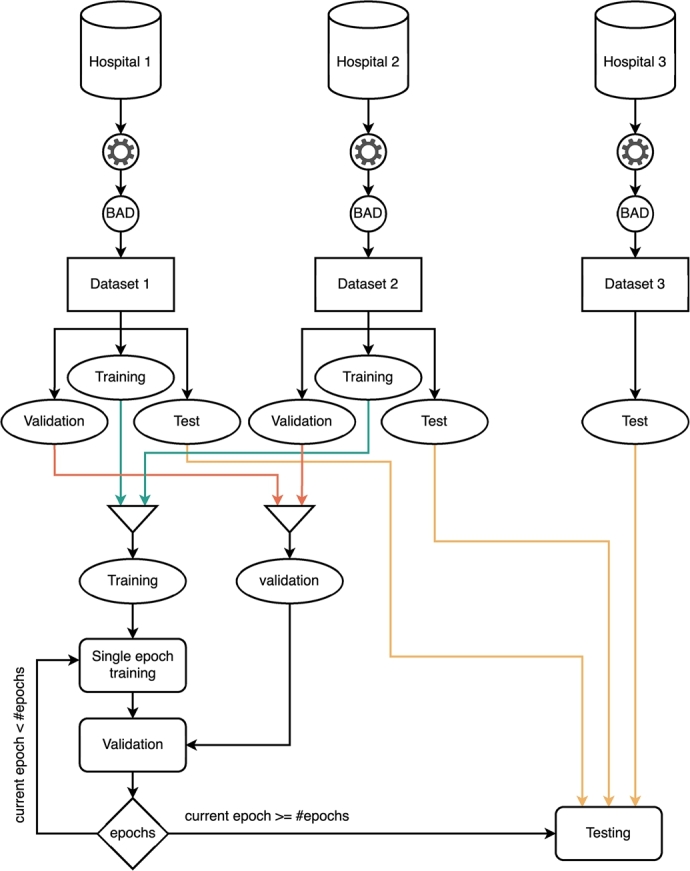
Dataset split per institution. This split is used on all the experiments.

An initial dataset was created and utilized as described in Fig. 2, which highlights three main areas: a) data collection and processing, b) data combination and splitting, and c) the training procedure. The processing mechanism, which will be described in detail in Sections 3.2 and 3.3 is applied independently to each hospital's data, resulting in the creation of Dataset 1, Dataset 2, and Dataset 3.

The dataset creation process is shown in Fig. 2. Each of the first two datasets is split into training, validation, and test sets following the percentages 70%, 20% and 10%, respectively. Although these sets contain standalone mammograms without any information about the corresponding patients or related mammograms, the split is performed on a patient-wise basis. This ensures that no information leaks from the training set into the validation and test sets. The training and validation sets are then combined into a single final training set and a single final validation set, respectively. The test sets, however, are kept separate, as it is crucial to evaluate the model's performance for each data provider individually. Due to its limited number of images, Dataset 3 is not included in the training procedure. Instead, it is converted into a single test set to evaluate the model's performance specifically on this hospital's data.

**Fig. 3:**
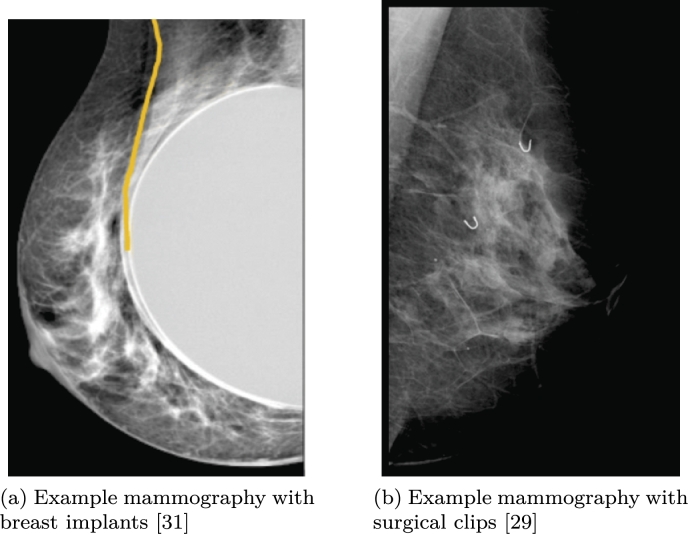
Examples of images with artifacts which affect model performance. Breast implants (left) introduce a foreign body which confuses the model. Surgical clips (right) are depicted as white small spots with may be confused by the model as abnormalities.

After the observation that the performance of the model in the initial FL experiment was suboptimal (more details in Section 4.3), the datasets were re-evaluated in consultation with the medical professionals from each hospital to identify whether the issue is data-related. In particular, we found that 6 patients had breast implants and 11 were not correctly scanned. Therefore these were removed. Moreover we found that there were several issues such as some images including labels on them, some of them were scanned and some had surgical clips in the depiction. It should be noted that such artifacts are not uncommon in mammography images [29]. These differences did not affect the classic training as when all images are put together there are more of these samples, but it affected greatly in FL. From the two hospitals, the one that had more variability in these images was Hospital 1, the one whose F1 score was lower. In fact, Hospital 1 included images from smaller hospitals, therefore including more variability on the machinery parameters () used to take the images, which can lead to trouble when creating a classifier as seen in Killim et al. [30]. In addition, it was identified that images between hospitals 1 and 2 had significantly different pixel intensity ranges, which was taken into consideration to improve FL model performance. Fig. 3 presents some examples with imaging artifacts which would affect model performance and were removed.

Finally, medical professionals indicated the need to remove any images labeled BIRADS 0 or 6, as these labels are considered noisy and would add confusion to the model. The final number of images per BIRADS type is the one that can be seen in Table 1.

**Table 1:** Final count of images for each hospital and BIRADS.

BIRADS	H1 (Gr)	H2 (Se)	H3 (Gr)	Total
0	0	0	0	0
1	30	10	0	40
2	212	297	21	530
3	0	33	0	33
4	136	43	6	185
5	112	72	22	206
6	0	0	0	0

**Total Images**	490	455	49	994
**Total Patients**	132	145	17	294

## 3 Materials and methods

In this section we provide details on the methods used. In particular we first describe what FL is and then we briefly introduce our framework to perform FL. After that, all the details regarding the pre-processing are given, including the Breast Area Detection (BAD) pre-processing step.

### 3.1 Federated Learning

**Fig. 4:**
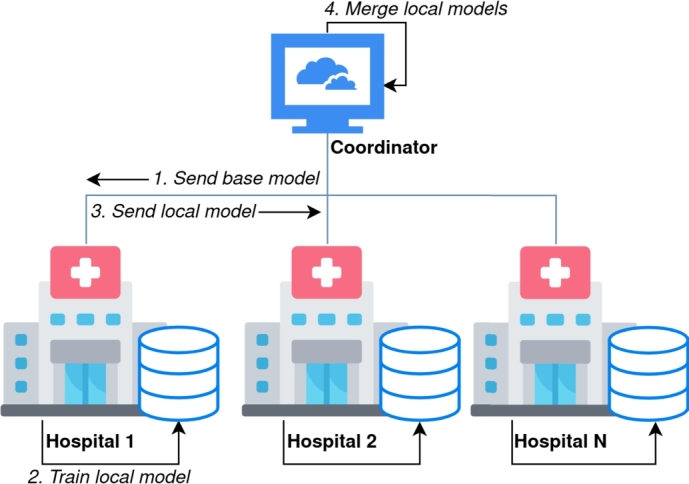
FL process. The process starts with the server providing a base model to every client. Then the clients train their local models. Finally, all the models are merged. Then, if a new round starts, the merged model is again sent to the clients to restart the process.

 is a way of training  models without having to move the data from where it is originally stored. Instead, the model is trained where the data is located, i.e., the hospitals, and then combined centrally. Fig. 4 represents this training schema. The FL server is the coordinator of the training procedure and the one that merges the models that are produced in the FL clients. The FL clients are the pieces of software that run in the premises of the hospitals, where the data is stored. In this way, the data does not have to leave the premises of the hospital, avoiding unnecessary data transfers to machines that might not be trusted.

The process works as can be seen in Fig. 4. First, the FL server requests a training process to be performed, then the FL clients train on their data and return the model to the FL server. Then, these models are aggregated (e.g., averaged). By doing so, if the process is correctly configured, the resulting model summarises the training performed by the clients. All this process is considered an **FL round** and can be repeated several times to ensure the quality of the model. After all the rounds, the final model is served by the FL server.

In this work our setup follows the FedAvg merging function [31]. This function takes all the model weights (which is known as model generally) and averages them with all the weights provided by the all the clients/hospitals. Although there are existing frameworks such as Flower [32], usually they lack of integration with modern resource managers such as Kubernetes, which is based in containerized applications that can be run wherever is needed. Therefore, to cover that gap we designed a language agnostic Kubernetes based framework. The main idea of our framework^4^ is to enable the developer to code in whatever language they want and then communicate through a standard Application Programming Interface (API). This procedure has the advantage that the system administrators of each hospital have just to set up a machine with an operative system compatible with Kubernetes and provide basic information the federation IT manager. This setup brings centralized control of the software deployed while releasing the IT from the hospital from maintaining and correcting most of the errors in the service.

**Fig. 5:**
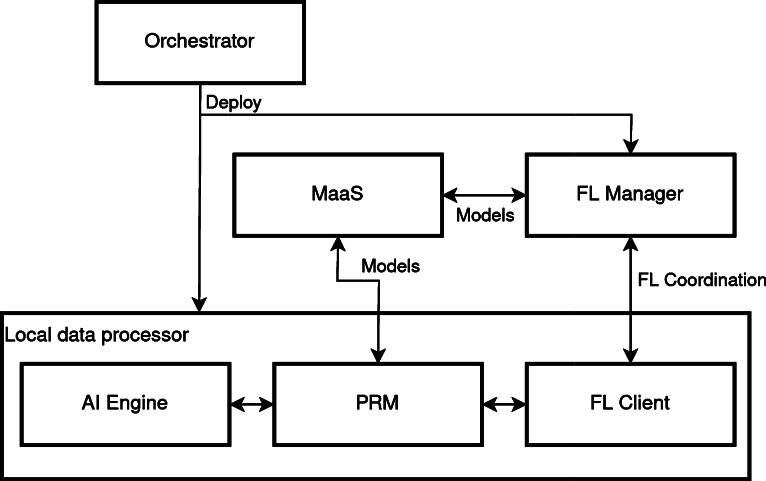
Federated Learning framework diagram.

As shown in Fig. 1, this infrastructure is actually distributed. This means that there is a coordination central cluster and then the different local clusters/computers located at the hospitals' premises. Fig. 5 shows the main components of the framework. First of all, in the centralized cluster we can find the *Orchestrator*, that is in charge of managing all the other components. Then, the  hosts all the models and all intermediate data that is required to be stored. Those two components are static in the infrastructure and always present.

On the other side, the *FL Manager* is deployed on demand. This means that when a training procedure is launched, the *Orchestrator* deploys this component to manage the process. Then, the *Orchestrator* deploys all the components of the *Local data processor*, however this is not deployed in the central infrastructure but in all the cluster/computers located at the hospitals' premises. This allows us to have components that are able to train models with the data stored in the local repositories.

As seen in Fig. 5, there are three components in the *Local data processor*. First, the *FL Client*, in charge of managing the local training and coordination with the central manager. Second, the  which is in charge of managing all the resources and architecture specific processes to make the interaction with the framework more transparent to the user. And third, the *AI Engine* which contains the code that the developer builds. The *AI Engine* can be programmed in any kind of language as long as it offers an HyperText Transfer Protocol (HTTP) API that is compliant with the specifications.

#### 3.1.1 Adapting code to the framework

As our framework works with an API as main entry point for the code and it is agnostic of the language and tools used to build the previously mentioned *AI Engine*, the effort on adapting the centralized code to the environment is minimal. The modifications are the following:

• Create an API endpoint that can be pinged to check if the initialization has finished and it is ready to receive requests
• Create an API endpoint to run the different use cases
• Include the functionality to merge models
• Include the functionality to train from an already trained model (initialized weights)

Notice that the different use cases needed to be implemented are the following: train a new model, train a model that is pretrained, evaluate the model, merge models and perform inference. Even though it is technically easy to adapt the code to the framework, there are new details that have to be taken into account, like new parameters such as the number of FL rounds to perform. By default, the merging function is provided by the framework if the user makes use of *Pytorch*, but can be customized by the user. Implementing both training and merging allow users to perform whichever operation or use any model type (e.g., random forests) they want instead of having one set of default type of models.

### 3.2 Image pre-processing

The DICOM files for the mammograms of the dataset contain essential information not only for the technical aspects of the image but also for the content of it. Thus, specific DICOM attributes, described in Table 2, are utilized for the pre-processing steps.

**Table 2:** The DICOM codes utilized for the processing of the digital mammograms.

Code	Description	Values	Example Values
(0028, 1052)	Rescale Intercept Attribute	Decimal String	-1024, 0, 00
(0028, 1053)	Rescale Slope Attribute	Decimal String	1, 01, 0.001
(2050, 0020)	Presentation LUT Shape Attribute	Code String	IDENTITY, INVERSE
(0028, 1041)	Pixel Intensity Relationship Sign Attribute	Signed Short	-1, +1
(0028, 0004)	Photometric Interpretation Attribute	Code String	MONOCHROME1, MONOCHROME2
(0028, 0030)	Pixel Spacing Attribute	Decimal String	[0.703125, 0.703125]
(0020, 0062)	Image Laterality Attribute	Code String	L, R

DICOM code pairs (0028, 1041), (0028, 0004) and (2050, 0020) should be reviewed to determine if the pixel values need be inverted. In particular, if Pixel Intensity Relationship Sign equals to 1 or Photometric Interpretation Attribute is set to “MONOCHROME1” or Presentation LUT Shape Attribute is assigned the value “INVERSE”, then each pixel gets inverted by subtracting its value from the maximum pixel value of the image.

At this second step, the pixel array should be horizontally flipped to align with the predetermined laterality, thereby eliminating an additional factor that could introduce bias into the model. Technically, the Image Laterality Attribute (0020, 0062) is inspected and if it is assigned the value “R”, the image will be flipped. In this way, the area of interest (the breast area) will always be located at the left side of the pixel array.

As mammograms are used to detect very small lesions in screening, they can be of high resolution such as 3328 × 4096 pixels [33]. However, these sizes make the training procedure really challenging from the perspective of computing capacity. Thus, having computational constraints due to limited resources, it is commonly accepted to use reduced resolution images for feeding Machine Learning model during the training process. Aiming to achieve a proper resizing to the images, it is crucial to engage the Pixel Spacing Attribute (0028, 0030) for preserving spatial relationships, maintaining accurate measurements, sustaining consistency across different devices and resolutions and improving image analysis accuracy.

### 3.3 Breast area detection

It is not rare to find printed labels on mammograms, especially when talking about digitized versions. Digital images are usually clear but, even in such cases, labels or annotations may exist. These labels are mostly related to the laterality of the breast or the view of the mammogram. Such elements can potentially influence the learning capability of the model during the training procedure; not only they tend to add another bias to the dataset but, also, the extraction of the actual region of interest is getting much more difficult.

To address this problem, we introduce the *Breast Area Detection (BAD)*^5^ tool which takes as input a mammogram and generates a cleared version as output, removing the useless information by the initial image. It consists of three main parts; a) the unsupervised breast area masks generation, b) the data augmentation and c) the supervised breast area mask generation.

#### 3.3.1 Preparing the BAD dataset

Two auxiliary datasets have been generated for training and evaluating the BAD tool; a) the Unsupervised Learning Dataset (ULD) and b) the Augmented Unsupervised Learning Dataset (AULD). For the purposes of this task, the INBreast [34] mammogram dataset is utilized. It includes 410 digital mammograms that correspond to 115 cases, 90 of them are women with both breasts affected, while the remaining 25 women have undergone mastectomy.

**Fig. 6:**
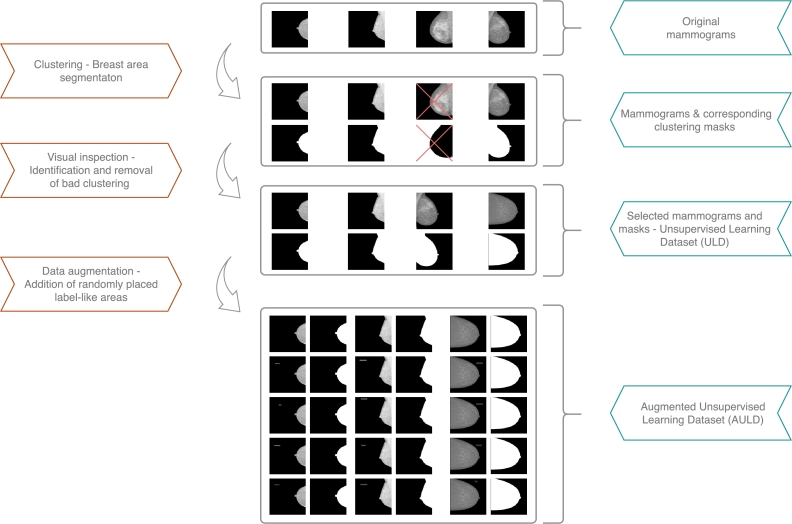
Pathway from the initial INbreast dataset to the finalized AULD dataset.

These images are the inputs to the K-means clustering algorithm that generates the initial breast annotation masks. Due to the uncertainty introduced by this method, the produced annotation masks are visually inspected in order to select only the proper ones. The output of this selection task refers to the ULD. In an aim to generalize the capacity of the tool, the idea of a supervised segmentation model is introduced. Towards this direction, the idea of augmenting this initial dataset (ULD) is emerging due to a) the limited amount of data (less than 410 images since we have eliminated some after the clustering procedure) and b) the demand to generate randomly sized and positioned boxes simulating, in this way, the undesirable labels. For each selected mammogram, five variations are used for the final augmented dataset; the first corresponds to the original one while the rest correspond to copies of the original including a randomly-sized and randomly-placed, fake label. This route from the original INbreast dataset to this final AULD is depicted in Fig. 6. Eventually, this approach pushes the UNet model to learn detecting the area of interest (breast area) in a more targeted way. At the same time, the generated dataset contains more than 1500 images which are considered to be sufficient for the training of the BAD model.

#### 3.3.2 Training procedure and usage

The training procedure is straightforward since the dataset is clear, targeted to the areas of interest and, thus, the hyper-parameters fine tuning is considered an easy task. 1000 images are used for the training of the model, 300 for the validation at the end of each epoch and the rest for the test set to practically evaluate the performance of the final model. Table 3 contains comparative evaluation of BAD with other similar tools in the literature. Even though its performance is slightly worse than SAM-based tools [35], [36], the computational complexity of SAM is significantly higher. The added computational complexity does not warrant the small performance increase.

**Table 3:** Comparative performance evaluation of BAD on the Inbreast dataset.

Model Name	Dice Score	IoU
Thresholding [37]	94.70	90.06
MedSegDiff [38]	98.37	96.82
SAM [39]	92.04	85.65
SAM-adapter [35]	99.05	98.13
SAM-breast [36]	99.27	98.55
BAD	98.48	97.01

**Fig. 7:**
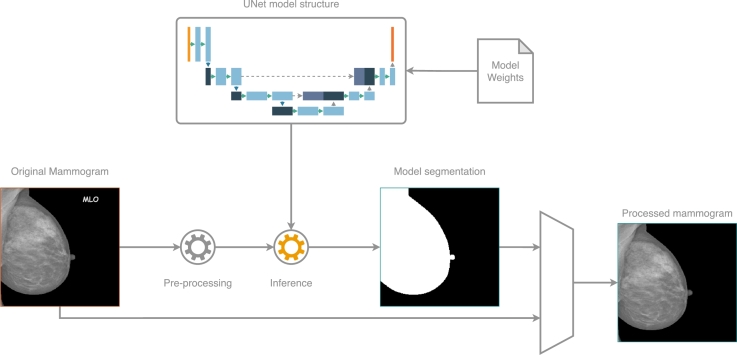
The BAD tool within the image processing pipeline, as described in Section 3.2.

After training the UNet model, the BAD tool's inference can be utilized as a standalone component within the processing pipeline. As shown in Fig. 7, the basic pre-processing methodology is applied to the original mammogram, and the output is then passed to the BAD tool's inference mechanism. The model's prediction, in the form of segmentation, is combined with the original mammogram to generate a cleaner version. Further processing can be applied to this final output by performing simple peripheral cropping, which is feasible since there are no labels or other artifacts. This results in a reduction of unnecessary information.

**Fig. 8:**
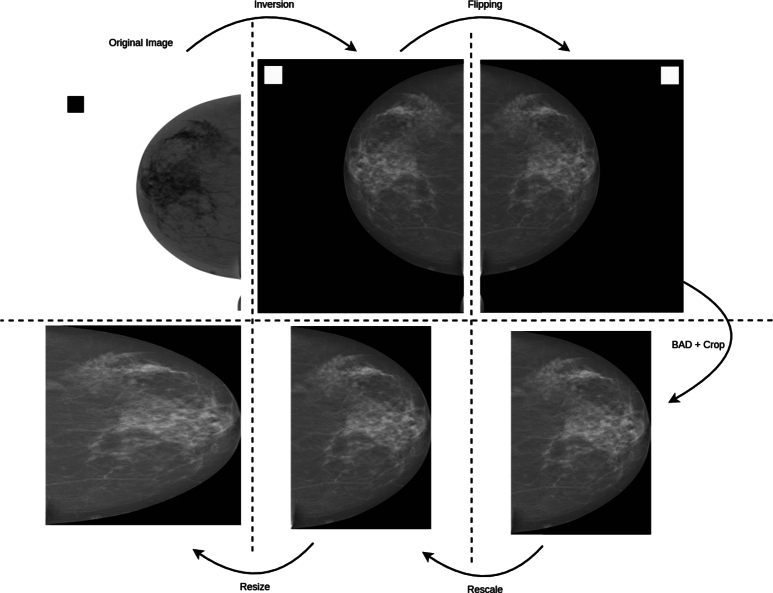
Visual representation of the impact of all pre-processing of an example image from the Inbreast [34] dataset. The image was contrast-inverted for demonstration purposes.

Fig. 8 presents a visual overview on the impact of all pre-processing steps on an example image. After potential inversion and clipping, the image is resized to 512x512 to be given as input to the BAD classifier. The BAD output is then used to crop the image so that the background area is minimized, as it does not provide any meaningful information to the classification model. Finally, the resulting image is resized to 256x256 to serve as input to the BIRADS classification model.

## 4 Results

### 4.1 Experimental setup

**Fig. 9:**
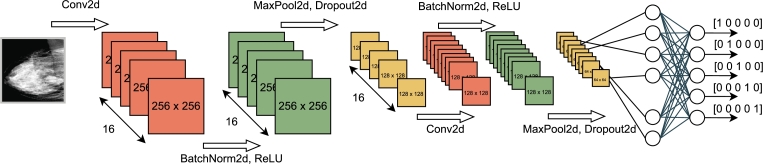
The structure of the model used for BIRADS classification.

To evaluate our framework on the BIRADS prediction model training procedure, we performed a two-step experiment. First, the model is trained in a centralized/classic manner, where all data are gathered in one place. We then performed model training using Federated Learning, where the data is distributed into separate nodes/hospitals, to compare both approaches. Information about the data and the splits is detailed in Section 2.2. The model architecture is based on Convolutional Neural Networks (CNN), as shown in Fig. 9. We have used convolutional layers since they do not require a large number of parameters and, thus, they do not create a communication bottleneck when transferring the models on each FL round.

The objective is to quantify how well do the trained models perform over the same dataset with a similar configuration. The only parameter that varied from model to model was the number of epochs, i.e., number of times that the model “sees” the complete dataset. In the central version, we have used 100 epochs, whereas in the federated version we have used 3 different combinations: 100 rounds - 1 epoch, 50 rounds - 2 epochs and 20 rounds - 5 epochs.

To perform the experiments we used the two infrastructure types presented in Section 2: a) a centralized repository containing all the required data and b) a federated infrastructure. The federated infrastructure main server was hosted in the same centralized cluster, whereas the clients were hosted in a machine located in each hospital premises (Greece and Serbia) to test how a real life environment can work, including network delays, difference of computing power and other issues that might happen on a complex setup like this.

### 4.2 Classic experiment

At this stage, a normalization procedure is applied to all sets based on the minimum and maximum pixel values of the training set. The training set is divided into batches and fed into the model for a single epoch of training. Following this, an evaluation process is conducted over the batches of the validation set. Subsequently, a decision is made according to the following algorithm: if the current training epoch has not exceeded the predefined total number of epochs (100 in this case), the procedure (single epoch training and validation) is repeated. Otherwise, the training is stopped, and the model is evaluated on the test sets. Table 4 presents the set of hyperparameters used during model training.

**Table 4:** Best hyperparameter set found in the initial experiment.

Hyperparameter	Value
Loss function	Cross-entropy loss
Learning rate	10^−8^
Optimizer	Adam
Batch size	10
Epochs	100

### 4.3 Adaptation of the problem to Federated Learning

#### 4.3.1 Initial adaptation

With previously mentioned model hyperparameters, several experiments were performed regarding the FL, mainly the trade-off between local epochs and FL rounds. The optimal choice in this case was 50 FL rounds with 2 local epochs. To adapt the classic version, the code was modified slightly to adapt it to the FL framework, however only communication parts were modified, the training part was untouched.

**Fig. 10:**
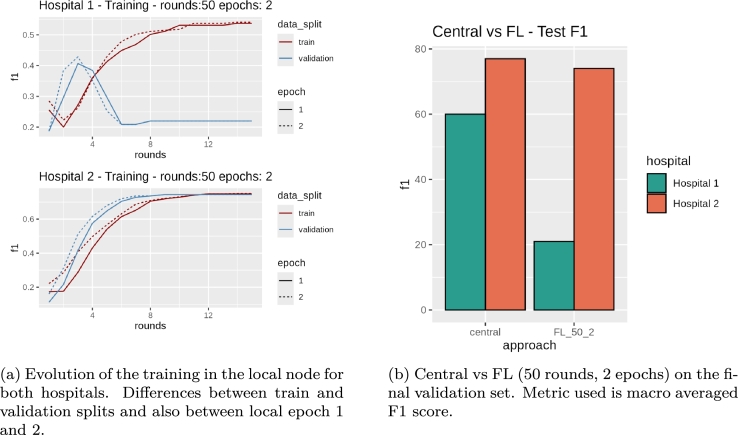
Model performance per hospital during the initial FL adaptation.

As can be seen in Fig. 10b, the F1 score for the Hospital 2 (in orange) is usually higher and that happens both in the central and the federated experiments. Notice that the score is slightly lower in the federated experiment, which is expected in FL settings. On the other hand, Hospital 1 (in teal) suffers from a serious decrease in performance compared to the classic experiment (∼40% decrease in F1). As we can see in Fig. 10a, from round 4 the model drifts away from the test set for Hospital 1, which means that there are very different images that are not accounted in training for Hospital 1 or for Hospital 2, as in each round the model is synchronized. Therefore we need to go deeper to understand what is happening.

#### 4.3.2 Improved pre-processing adaptation

**Fig. 11:**
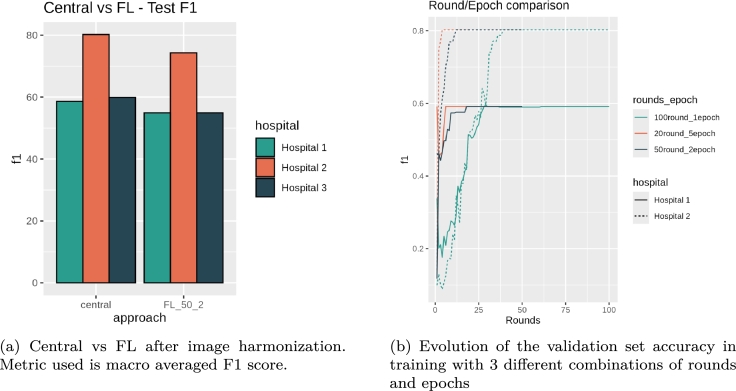
Results of the final experiment including the whole preprocessing pipeline.

After the preprocess, the FL training was performed in the same settings. Notice that only Hospital 1 and 2 are used for the training as in the previous experiment. We include Hospital 3 only to be used in model testing as seen in Fig. 2. As seen in Fig. 11a, performance of both, the classic experiment and the combination of 50 rounds and 2 epochs is increased with the full preprocess. The performance of the new Hospital 3 (dark blue) is similar to Hospital 1, showing generalization of the approach. However, the same happens when we analyze the rest of round/epoch combinations. Again, we tested 3 different combinations: 100 rounds - 1 epoch, 50 rounds - 2 epochs and 20 round - 5 epochs. This means that in general we will do 100 epochs in each combination. As can be observed in Table 5, in this particular problem, all the combinations converge to similar solutions. As seen in the table and figure, now Hospital 1 accuracy is not far from the classic version. In particular, Fig. 11b shows the test F1 score while training for all combinations. Now Hospital 1 does not fall in overfit. The fastest solution was the 50-2 combination as seen in Table 5. However, this is because the infrastructure is being shared and is dependent of the network and machinery from other institutions. Usually, the fastest approach should be the 20-5 as it requires less communication between the nodes.

**Table 5:** Results of the hyperparameter search on the trade-off between Rounds and Epochs, showing the F1-score for each hospital. The 3 solutions are almost identical as the model converges to similar weights.

Rounds	Epochs	Hospital 1	Hospital 2	Hospital 3	Train time (min)
100	1	0.549	0.743	0.559	260
50	2	0.550	0.743	0.559	192
20	5	0.549	0.743	0.559	247

## 5 Lessons learned and recommendations

 is a very interesting research topic that promises benefits for situations in which is impossible to share the data. However, it cannot be seen as a plug and play solution, since many details needs to be carefully considered to ensure successful implementation. In this Section, we summarize important lessons learned, and propose mitigation actions for specific encountered problems.

• **Inhomogeneous data leads to inaccurate models:** given the nature of data, data from different providers can be different, as can be seen from our case study as well as other works [30]. Variations might not affect a human radiologist, but they might have a big impact on the accuracy of the models produced. This fact is accentuated in FL, as we are building models independently in each Data Provider, we might find a situation in which the models do not converge or that are biased towards some concrete population. Data homogeneity may also directly impact FL hyperparameter tuning, as the local model may overfit the respective provider's data, thus impacting model aggregation and hampering its generalization capabilities [40].
  **Mitigation actions:** Standardized data collection protocols across data-sharing institutions should be established to ensure consistency in data formats, imaging modalities and annotations. These protocols should be enhanced through data annotation procedures and guidelines, which should be commonly-defined by medical professionals. Finally, each hospital/medical institution should report dataset metadata (e.g. scanner type, acquisition settings) alongside images to enable model adjustments for potential domain shifts.
• **Data should be homogenized:** following the previous point, harmonization is the most important part of AI medical service development. One of the main challenges that the AI developers face during model development is the high degree of harmonization required between different Data Providers. One cannot expect that Data Providers from different institutions will use the same equipment for data generation. This fact is explored by Kilim et al. [30] where they explore the parameters from different imaging machines and different hospitals. They saw that there is a clear difference between images from different centers and used the parameters from the machines to improve the accuracy. Therefore, harmonization of medical images between *Data Providers* is one of the most important steps in the development pipeline, and can “make or break” the effectiveness and generalization capabilities of a model. Following Kilim et al. [30], other important step to do is to keep the image metadata regarding the machinery used. This metadata can be used for the harmonization or afterwards for the pre-processing of the data in the FL environment.
  **Mitigation actions:** The researcher shall ensure the standardization of pre-processing pipelines across all participating institution. On a communication level, information exchange between nodes (e.g. normalization values, dataset size etc.) should be enabled. In addition, the researcher should investigate the utilization of data augmentation and harmonization techniques to reduce variability. Finally, the regulatory bodies are encouraged to define minimum dataset standard for FL participation (e.g. image resolution, labeling consistency).
• **The data should be curated prior FL training:** even though Federated Learning is about securing the data by not showing it to the developers, in the end developers need to have a glimpse on the data to understand how to work with it and to debug the models. This could be done with aggregations that fulfill the privacy requirements (e.g., anonymized data with K-Anonymity guarantees). Another option is to provide a representative sample of the data that can be shared (anonymous). Finally, it is important that the *Data Providers* collaborate through a figure such as a *Data Manager/Curator* that can check particular pieces of information for the developer.
  **Mitigation actions:** We firmly believe that the *Data Manager/Curator* will be one of the most important occupations of the future. In medical imaging FL, each hospital/institution shall assign a Data Manager, who will be responsible for dataset curation, compliance with annotation/data collection protocols, as well as be the main contact point for data-related questions from researchers. In addition, a small, yet representative dataset sample shall be anonymized or even generated synthetically from the original data and made available to researchers to gain a visual understanding of the underlying data. An alternative would be to introduce data access mechanisms such as Federated Analytics that are specific and have institution-tailored privacy requirements (e.g. K-anonymity guarantees).
• **Federated Learning alone does not guarantee privacy preservation:** FL was born from the necessity of training models without sharing the data. This is a perfect fit for healthcare applications as patient data is very sensitive. However, take into account that to set such system you need an expert on the topic as there are many aspects to cover. The naïve FL we followed [31] was good enough for our purposes and the “safe” environment created through the various data sharing agreements that all involved parties signed and committed to. However, it is not considered secure in most scenarios as you need to assume that there are no attackers on the client side nor in the server side. If it is not possible to assume this, security measures need to be put in place, such as secure aggregation and attack detection. However, these kind of measures work on detriment of the model accuracy as usually the measures imply processes such as adding noise to the original data to avoid leakages. Finally, as the complexity of the model grows, the ability to memorize data increases. Even though the process is secured, the final model should be tested against information leakage attacks such as the ones that can be performed on *Diffusion models* [41]. Notice that this is not the case of our model as it is not a complex network, but it should be always checked as there is no rule of thumb to measure when a model might leak.
  **Mitigation actions:** On a security level, the models shall be made robust against attacks. Secure aggregation and adversarial attack detection are necessary to ensure model robustness. In this line there are approaches such as KRUM [42] to avoid specific attacks which can be used along our methodology. Continuous tests against information leakage from model weights are encouraged [41]. Finally, on a regulatory level, the hospitals/institutions shall conduct privacy impact assessments before joining FL networks.
• **IT personnel and Hardware must be considered in real scenarios:** FL in a real scenario usually hides two complexities: The complexity of the implementation itself and the disparity of resources. The first problem is that the solution, even though it will be functional, it might be hard to understand for the IT personnel required present in the particular institution that wants to be involved in the federation. The second occurs when one of the institutions involved in FL has less computational resources than the others. In the case of regular FL all the institutions will be waiting for the one that has not yet finished the round. Finally, network might also be an issue when doing FL as in each end of round, the nodes must be synchronized. In this step, the partial models will be sent to the FL coordinator to produce the merged model. This is problematic when large models are trained.
  **Mitigation actions**: To alleviate the work required by the IT personnel from each institution, the work must be designed from the users and maintainers perspective. This means that the framework needs to be easily maintainable for persons without specific knowledge in FL. In the case of our framework, the IT personnel only required to provide a machine that matched the minimum requirements with a working network connection. All the management of the software was centralized through Kubernetes and KubEdge so that it could be easily maintained by one system administrator involved in the work. Regarding the disparity of resources, if there is no possibility of homogenizing the available resources we recommend to apply asynchronous FL such as the work of Li et al. [43]. Notice that if there are similar resources but one node is taking more to compute it might also be because of the number of data points in that particular node or even the processing that has to be done over those nodes (e.g., images that must be uncompressed internally in the code to be able to use them). Finally, the model size must be check to see if it is viable to send this information through the established network. If an estimate of the communication is provided to the IT personnel they might be able to help on the structural part.
• **Network is related to the trade-off between Federated Learning rounds and local Epochs:** The tradeoff between FL rounds and local epoch is that the more rounds (with less local epochs) are done, the better is the result. This is because all the models are synchronized more frequently, avoiding overfitting on local data. This is very important in scenarios where non- data is present. However, this also increases the amount of communication that has to be done and it will have an impact on the training time as multiple institutions are interconnected with different network capabilities and availability.
  **Mitigation actions:** Before performing a full-fledged study on the hyper parameters, it is important to understand the communication that will occur and how much amount of data has to be transferred through the network, as mentioned in the previous lesson learned. If possible, start doing the study with a small portion of data just to quantify the amount of communication that will happen with the different number of rounds and epochs. If the network does not have a big bandwidth or the models are big (order of GBs), we advise to enlarge the local epochs and reduce the FL rounds. In other cases, we advise to go for one local epoch and as many FL rounds as needed. Nevertheless, it is advice to test more than one scenario to study the convergence of the model. This has to be performed jointly with the regular hyperparameter tuning.

Table 6 summarizes the aforementioned lessons learned and proposed mitigation actions. In addition, it links them to FUTURE-AI guidelines, which is an international, multi-stakeholder initiative for defining and maintaining concrete guidelines that will facilitate the design, development, validation and deployment of trustworthy AI solutions in medicine and healthcare [11].

**Table 6:**
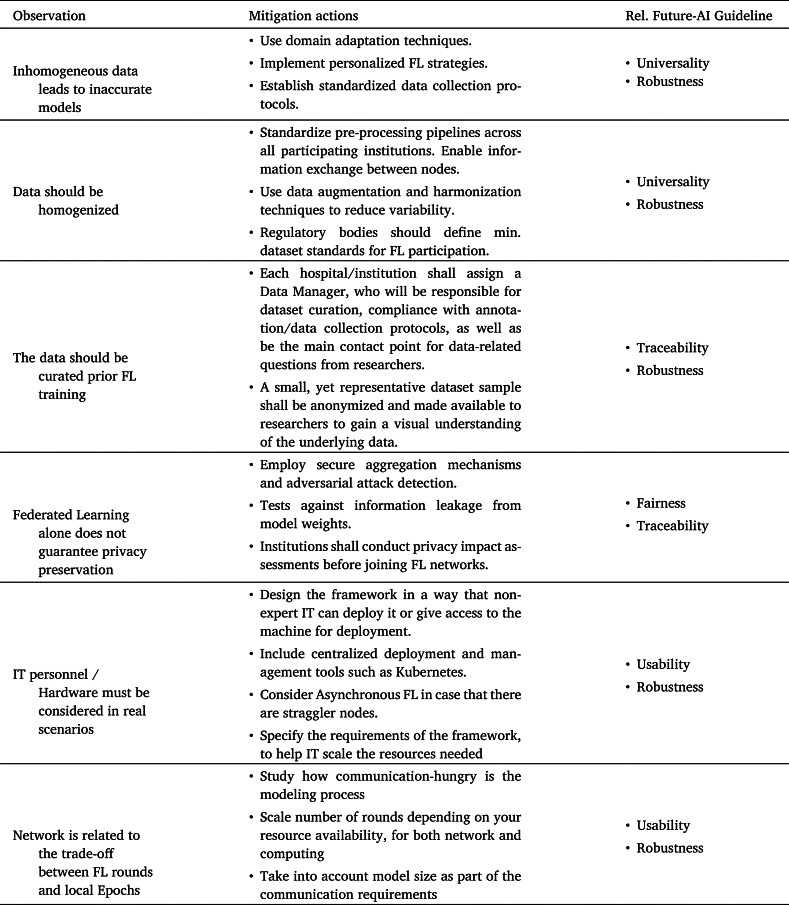
Lessons learned, mitigation actions and linked FUTURE-AI guidelines.

## 6 Conclusions

FL is a promising research area that enables training models without sharing the data, which is specially interesting in the Healthcare domain. However, adapting a classic centralized training procedure might not be trivial, specially when handling non- data. In this experiment we highlight the importance of harmonizing the data that is being used through image processing techniques, even if initially the classic training has no major issue using different data together. Of course, the main difficulty of this process is that in FL we are not able to directly access the data, therefore we need other type of mechanisms such as aggregated information provided using Federated Analytics, for example. On the other hand, there might be problems within the data that are not easily solvable, such as breast implants or labels that might rarely appear and are not annotated in the metadata. This kind of issue requires to have a control over the Hospitals' data that is not available and, therefore, the Hospitals should be aware of their data and help the researchers modeling their data through FL, not just provide computing power and data. Finally, we highlight the relevance of having as much homogeneous data as possible so that the result can be close enough to the classic approach.

## CRediT authorship contribution statement

**Ioannis N. Tzortzis:** Writing – original draft, Software, Methodology, Formal analysis, Data curation. **Alberto Gutierrez-Torre:** Writing – original draft, Validation, Supervision, Software, Methodology, Formal analysis, Conceptualization. **Stavros Sykiotis:** Writing – review & editing, Validation, Methodology, Data curation, Conceptualization. **Ferran Agulló:** Validation, Software, Formal analysis. **Nikolaos Bakalos:** Writing – review & editing, Writing – original draft, Supervision. **Anastasios Doulamis:** Supervision, Investigation, Funding acquisition. **Nikolaos Doulamis:** Supervision, Investigation, Funding acquisition. **Josep Ll. Berral:** Supervision, Investigation, Funding acquisition.

## Declaration of Competing Interest

The authors declare that they have no known competing financial interests or personal relationships that could have appeared to influence the work reported in this paper.

## Footnotes

2 https://incisive-project.eu/.

3 https://cancerimage.eu/.

4 https://github.com/INCISIVE-Training-Inference-Framework.

5 https://github.com/itzortzis/breast_area_detection.
